# Mapping the global scientific knowledge of peste des petits ruminants virus from 1990 to 2023: Identifying research hotspots, gaps, and future directions

**DOI:** 10.5455/javar.2025.l875

**Published:** 2025-03-24

**Authors:** Ibrahim M. Alzuheir

**Affiliations:** Department of Veterinary Medicine, Faculty of Veterinary Medicine and Agricultural Engineer, An-Najah National University, Nablus, Palestine

**Keywords:** Global trends, PPR, PPRV, research activity, small ruminants

## Abstract

**Objective:**

This study aims to analyze and map existing research literature on peste des petits ruminants virus (PPRV) to identify research hotspots, knowledge gaps, and future directions. PPRV causes peste des petits ruminants (PPRs) disease, leading to significant impacts on small ruminants through high mortality rates and trade limitations. Eradication programs are led by the Food and Agriculture Organization and the World Organization for Animal Health.

**Materials and Methods:**

Data were obtained from the Scopus database using keywords related to PPRV and PPR, covering the period from 1990 to 2023. Validation methods were employed to verify the search strategy’s accuracy. Data analysis focused on identifying temporal evolution, geographical distribution, key contributors, sources, research hotspots, and gaps, which were visualized using maps.

**Results:**

The study identified 478 research documents from 1,834 authors, with most being research articles (91.0%). A significant increase in publications was observed from 1990 to 2023, peaking in 2019 and 2021. India led with 112 articles, followed by the UK (79) and China (71). Top research institutions included the Indian Veterinary Research Institute and the Pirbright Institute in the UK. Major contributors like S. Parida and V. Balamurugan formed dense international collaboration networks. Key journals included “Transboundary and Emerging Diseases” and “Journal of Virological Methods.”

**Conclusion:**

This study reveals an increased global scientific production on PPRV, driven by international collaboration. However, research gaps remain, particularly from North African and Middle Eastern countries. Priorities include vaccine development, vaccination campaigns, veterinary capacity building, and enhanced reverse transcription polymerase chain reaction implementation. Insights from ths study can guide policymakers, funders, and researchers in prioritizing resources and strategies to eradicate PPRV, ensuring sustainable livestock health and economic stability.

## Introduction

Peste des petits ruminants (PPRs) is an acute, highly contagious, and often fatal disease caused by the peste des petits ruminants virus (PPRV), recently renamed as small ruminant morbillivirus (SRMV) [1]. PPRV belongs to the genus *Morbillivirus* in the family *Paramyxoviridae. *PPR primarily affects small ruminants (sheep and goats), leading to significant economic losses, particularly in developing countries across Asia, the Middle East, and Africa [2]. These regions often face inadequate resources for scientific research and control measures [3]. To combat PPR, international organizations, particularly the Food and Agriculture Organization (FAO) and the World Organization for Animal Health (WOAH), coordinate global surveillance programs that enable early detection and rapid response to outbreaks [4]. Collaborative research efforts focus on studying PPRV epidemiology, transmission, and control measures, which are crucial for developing effective vaccines [5]. Multiple international agencies fund PPRV research to optimize resources and prevent duplication [6]. Collaborative scientific publications disseminate findings, propelling innovation, and showcasing global research productivity and trends for strategic alliances in PPRV and PPR research [7].

Evaluating the current state of research on PPRV and PPR supports efforts to initiate projects, find collaborations, and secure financial sponsors [6]. This study aims to provide a descriptive assessment of the global scientific literature on PPRV and PPR. It evaluates the emerging trends of scientific knowledge and contributors and addresses the hotspots and research gaps in PPRV and PPR research. By examining the research landscape, policymakers and funders can gain insights into areas needing increased investment and support, ensuring that resources are efficiently allocated to address essential research questions. Additionally, understanding the current research networks and partnerships allows researchers and organizations to identify potential collaborators and promote interdisciplinary collaborations, ultimately aiding in the global effort to eradicate PPR.

## Materials and Methods

### Ethical approval

The research did not involve human participants or animals and utilized publicly available datasets; ethical approval was not required. All the data presented in this manuscript is available in the Scopus database (www.scopus.com) using the search query listed in the methodology section.

### Data source and assembly

In the current study, data were retrieved on July 15, 2024, from the Scopus database (www.scopus.com). Scopus affords basic and advanced search options. Its content covers well over 75 million records and >23,000 golden impact journals worldwide, including open-access journals [8]. All documents published in Scopus have an English title, abstract, and keywords; allow publication searching without a language restriction. Besides, Scopus allows retrieval of documents from other database sources, e.g., PubMed [9].

The search query was adopted after reviewing both scientific publications to set relevant keywords to be used in the search [10,11]. In this study, the search strategy was based on a search title, abstract, and keywords. Search results were restricted to the following Boolean query, executed within a single search of assembling two different search strings, one for each category (PPRV and the PPR disease), and combining these. For the virus, the Boolean operator (OR) is joined by the peste des petits ruminant virus OR PPRV OR SRMV, and by the Boolean operator “AND” to obtain only the intersection with the disease: PPR, or peste des petits ruminants. The complete search query was (title-abs-key (peste and des and petits and ruminant and virus) or title-abs-key (PPRV) or title-abs-key (small and ruminants and morbilli and virus) and title-abs-key (PPR) or title-abs-key (peste and des and petits and ruminant).

### Inclusion and exclusion criteria

All the resulting publication records were imported into a bibliographic referencing tool and manually assessed for relevance, removing articles that did not contain information relating to PPRV. The validation process ensured the absence of false positive results. Each article was confirmed to address the research topic and meet the study objectives. No false positives were found among these articles, indicating an accurate search strategy. The resulting articles were manually reviewed. Publications indexed either as conferences, editorials, posters, or errata were removed. The EndNote program was used to check for article duplication [12]. The retrieved publications were closely looking at the temporal evolution of publication, geographical location, countries, international research collaboration, institutions, authors, research theme, and journal source. The visualization of similarities viewer (VOSviewer) program was used to construct the visualization maps and assess the author’s collaboration and research theme [13].

## Results

The search query implemented in the Scopus database retrieved 479 research documents from 1,834 authors. Only one article was excluded after duplication, resulting in 478 research documents included in this study. The majority of the retrieved were research articles (*n =* 435; 91.0%), review articles (*n =* 32; 6.7%), book chapters (*n =* 9; 1.06%), books (*n =* 1; 0.21%), and short surveys (*n =* 1; 0.21%).

### Temporal evolution

**Figure 1.**
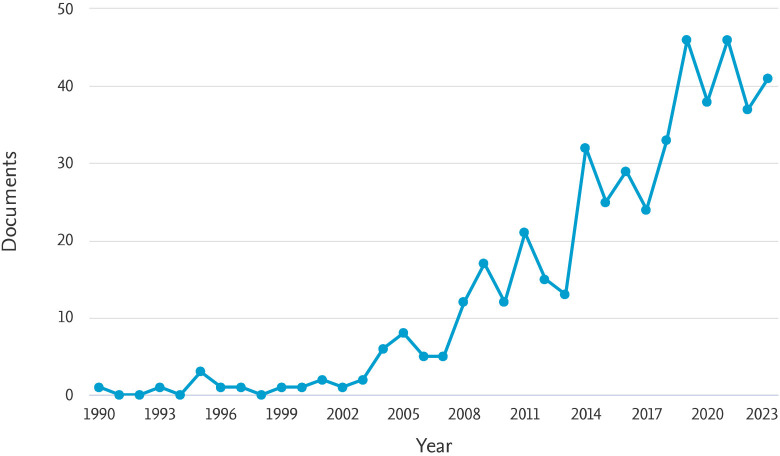
The number of publications on PPRV and PPR by year (1990–2023).

The temporal analyses of the 478 documents, originating from different sources published between 1990 and 2023, are shown in Figure 1. The annual scientific production shows a significant increase in research activity over the years. In the early 1990s, the number of articles published annually was quite low, with 1 article each in 1990 and 1993, and no articles in 1991, 1992, and 1994. A gradual increase in publications began in the mid-1990s, with 3 articles published in 1995 and a steady, albeit slow, rise in subsequent years. Notable jumps in the number of publications occurred in 2005 (8 articles) and 2009 (17 articles), indicating growing interest and research efforts in the field. A significant surge in publications is evident from 2011 onwards, with 21 articles published in that year. This upward trend continued, peaking in 2019 and 2021 with 46 articles each year. The number of articles remained high in the following years, with 37 articles in 2022 and 41 articles in 2023 (Fig. 1).

### Geographical distribution

The analysis of publications on PPRV and PPR from 1990 to 2023 revealed the top ten active countries in this research domain, as provided in Table 1. India emerged as the leading country with a total of 112 articles. The United Kingdom followed with 79 articles, and China ranked third with 71 articles. France contributed 51 articles, while Pakistan had 48 articles. Nigeria produced 26 articles, placing it in the sixth position. Austria was close behind with 25 articles. The United States had 23 articles, and Turkey contributed 21 articles. Ethiopia rounded out the top ten with 20 articles. Table 1 lists the top ten active countries in PPRV and PPR article production.

### Affiliation

The analysis of institutional contributions to PPRV and PPR research from 1990 to 2023 identified the top ten affiliation agencies shown in Table 2. The Indian Veterinary Research Institute in India led with 61 articles, highlighting its significant role in PPRV research. The Pirbright Institute in the UK followed closely with 59 articles. France’s French Agricultural Research Centre for International Development (CIRAD) contributed 48 articles, demonstrating its active involvement in PPRV studies. The Chinese Academy of Agricultural Sciences in China produced 37 articles. The Indian Council of Agricultural Research also made a substantial contribution with 36 articles, followed by Animal, Santé, Territoires, Risques et Ecosystèmes (ASTRE) in India with 35 articles. France’s Institut national de recherche pour l’agriculture, l’alimentation et l’environnement (INRAE) published 33 articles. The National Institute of Veterinary Epidemiology and Disease Informatics (ICAR) in India added 30 articles to the research landscape. The International Atomic Energy Agency in Austria contributed 25 articles, while Université de Montpellier in France rounded out the top ten with 23 articles.

**Table 1.** List of top ten active countries in PPRV and PPR (1990–2023).

Country	No. of articles
India	112
UK	79
China	71
France	51
Pakistan	48
Nigeria	26
Austria	25
USA	23
Turkey	21
Ethiopia	20

**Table 2.** Top ten affiliations agencies in PPRV and PPR articles (1990–2023).

Funding agencies	Country	No. of articles
Indian Veterinary Research Institute	India	61
The Pirbright Institute	UK	59
CIRAD^a^	France	48
Chinese Academy of Agricultural Sciences	China	37
Indian Council of Agricultural Research	India	36
ASTRE^b^	India	35
INRAE^c^	France	33
ICAR^d^	India	30
International Atomic Energy Agency	Austria	25
Université de Montpellier	France	23

^a^French Agricultural Research Centre for International Development.

^b^Animal, Santé, Territoires, Risques et Ecosystèmes.

^c^Institut national de recherche pour l’agriculture, l’alimentation et l’environnement.

^d^National Institute Of Veterinary Epidemiology And Disease Informatics, Bengaluru.

**Table 3.** Top 10 authors in PPRV and PPR article research (1990–2023).

Author Name	Institute	Country	No. of article
Parida, S.	The Pirbright Institute	UK	38
Balamurugan, V.	ICAR	India	35
Libeau, G.	CIRAD	France	29
Rajak, K.K.	ICAR	India	26
Mahapatra, M.	The Pirbright Institute	UK	23
Abubakar, M.	National Veterinary Laboratories	Pakistan	23
Singh, R.K.	Indian Veterinary Research Institute	India	22
Sen, A.	ICAR	India	21
Diallo, A.	International Atomic Energy Agency	Austria	21
Muthuchelvan, D.	ICAR	India	21

### Authors

The analysis of PPRV and PPR research from 1990 to 2023 reveals key contributors and their institutional affiliations as provided in Table 3. Leading the field is S. Parida from The Pirbright Institute in the UK, with an impressive 38 articles. Following closely are V. Balamurugan from ICAR in India with 35 articles and G. Libeau from CIRAD in France with 29 articles. This underscores the global distribution of expertise, with Indian researchers such as K.K. Rajak, M. Mahapatra, and A. Sen from ICAR contributing significantly with 26, 23, and 21 articles, respectively. International collaboration is evident through authors like A. Diallo from the International Atomic Energy Agency in Austria, alongside regional contributions such as those from M. Abubakar at Pakistan’s National Veterinary Laboratories. These findings highlight the diverse and collaborative efforts in PPRV and PPR research, crucial for understanding and managing this infectious disease affecting small ruminants worldwide.

### Author collaboration

**Figure 2.**
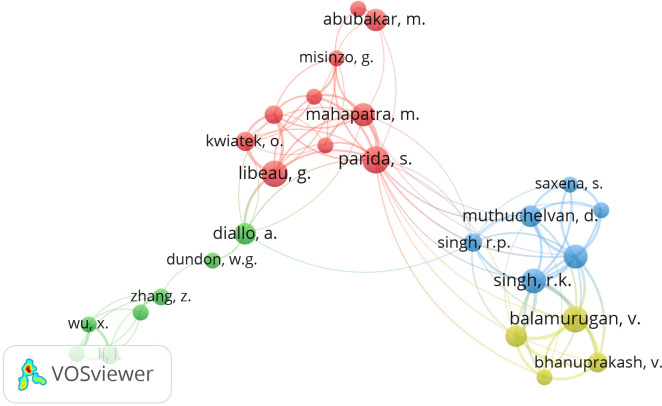
Network visualization map among authors with minimum research output of 10 documents on PPRV and PPR. The thickness of the connecting lines represents the strength of research collaboration between any two co-authorships.

The VOSviewer visualization provides a refined network of co-authorship among researchers working on PPRV and PPR studies (Fig. 2). This network showcases the collaborative relationships between various researchers, organized into several distinct clusters. Here is an analysis of this visualization; the red cluster is the most densely connected and includes key researchers such as Parida, S., Mahapatra, M., Abubakar, M., Misir, G., Kwiatek, O., and Libeau, G. The dense connections suggest robust collaborations and frequent co-authorship among these researchers, indicating a strong collaborative network. The blue cluster comprising Singh, R.K., Saxena, S., Muthuchelvan, D., Balamurugan, V., and Bhanuprakash, V. This group represents another significant collaboration network, highlighting their joint efforts in PPRV research. The green cluster includes researchers like Diallo, A., Dundon, W.G., and Zhang, Z. This cluster, although smaller, indicates specialized collaborations possibly focusing on specific aspects of PPRV and PPR research. The yellow cluster contains researchers like Balamurugan, V. and Bhanuprakash, V., suggesting another subset of collaborations, possibly region-specific. Key researchers in PPRV were Parida, who appears as a central node in the red cluster, indicating a pivotal role in PPRV research collaborations, and connecting with many other researchers. Mahapatra, M. was also central in the red cluster, suggesting a significant role in collaborative efforts. Singh, R.K. is central in the blue cluster, indicating his importance in this collaborative network.

### Research themes

**Figure 3.**
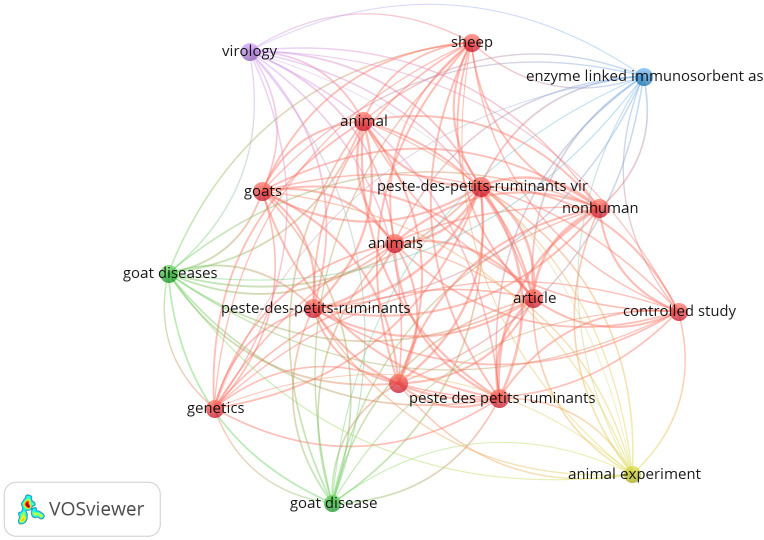
Network visualization map of most frequent keywords with more than 100 times occurrence in articles of the retrieved literature on PPRV and PPR (1990–2023). The five clusters represent the major 17 research themes in the retrieved literature.

The visualization from VOSviewer represents a network of keywords associated with PPRV and PPR (Fig. 3). Central keywords include Peste-des-petits-ruminants vir and Peste-des-petits-ruminants as central keywords and are highly connected emphasizing the virus and the disease it causes. The second cluster is the animal-related terms (“animal,” “animals,” “goats,” “sheep,” “goat diseases,” and “goat disease”) are heavily interconnected, indicating a strong focus on the impact of PPRV on various animals, particularly small ruminants. The hotspots of disease, virology, genetics, and enzyme-linked immunosorbent assay suggest a focus on the virological and genetic aspects of PPRV, as well as diagnostic methods. Research methods keywords such as “controlled study,” “animal experiment,” and “article” indicate the types of research and publications that are common in the field. The network shows strong interconnections between various animal-related terms and the main disease terms, highlighting the interdisciplinary nature of the research. The keyword “enzyme-linked immunosorbent assay” is somewhat peripheral but still connected to the main network, indicating its relevance in diagnostics but perhaps less central than some other topics. Some keywords like “controlled study” and “nonhuman” have fewer connections, suggesting they are important but not as central as other terms in this research area.

### Journal source

Research on PPRV and PPR from 1990 to 2023 has been extensively published across a range of journals as provided in Table 4. The “Transboundary and Emerging Diseases” journal led contributions at 7.95% of the total 478 articles. The “Journal of Virological Methods,” “Tropical Animal Health And Production,” and “Viruses” also feature prominently, each contributing over 3% of the total articles. These journals reflect a global distribution of research efforts, with notable contributions from Europe, North America, and Asia, as evidenced by journals such as “BMC Veterinary Research” and “Veterinary World.” This diversity highlights the multidisciplinary approach to studying PPRV, emphasizing the need for continued international collaboration to effectively manage and mitigate the impact of this infectious disease on global livestock populations.

## Discussion

The findings of this study provide a comprehensive analysis of global research trends on PPRV and PPR, offering fresh insights into this critical field. Notably, the significant increase in global research output from 1990 to 2023 highlights the growing recognition of PPRV as a major threat to small ruminant health and the global livestock economy. The steeper growth in research from 2019 acknowledges the importance of PPR as a threat to small ruminant livestock, resulting in the initiation of the FAO and WOAH PPRV Global Eradication Program aimed at eradicating the disease by 2030 [4].

PPR is considered to be a transboundary disease of great significance through its effect on the development and maintenance of sustainable agriculture in developing countries [14]. The current study indicated that research activity on PPRV was not limited to high-income, English-speaking countries. The geographical distribution highlights the global interest and research activity in PPRV and PPR, with significant contributions from both developed and developing countries. The predominance of India, the UK, and China underscores their leadership in the field and their ongoing commitment to understanding and managing PPR. Despite these advancements, critical gaps remain in PPRV research, particularly in regions such as North Africa, the Middle East, and sub-Saharan Africa, where the disease burden is highest [7]. Research on wildlife reservoirs, cross-species transmission, and regional variations in PPRV strains is limited, as is the study of vaccine efficacy in different climates and the real-world impact of vaccination campaigns [15]. Furthermore, diagnostic tools suitable for resource-limited settings and improvements in veterinary infrastructure in developing countries are underexplored. The reasons for research activity in the field of PPRV and PPR in different countries are multifaceted, involving factors such as the prevalence of PPR, research capacities, government support, international collaboration, and socioeconomic factors [14,16]. Addressing these gaps is essential for advancing PPRV control and global eradication efforts.

**Table 4.** Top ten journal sources in PPRV and PPR article research (1990–2023).

Article source	No. of articles	Country
Transboundary And Emerging Diseases	38	UK
Journal Of Virological Methods	21	Netherlands
Tropical Animal Health And Production	20	Netherlands
Viruses	16	Switzerland
BMC Veterinary Research	13	UK
Frontiers In Veterinary Science	11	Switzerland
Small Ruminant Research	11	Netherlands
Veterinary Microbiology	11	Netherlands
Plos One	10	USA
Veterinary World	10	India

The presence of distinct name patterns and affiliations suggests a wide geographic distribution of PPRV and PPR research, involving regions such as Asia, Africa, and Europe, indicating international involvement in PPRV research [17]. The network shows interconnections between different affiliations, indicating interdisciplinary and cross-institutional collaborations. The density of connections within clusters in Figure 2 highlights strong collaborative efforts and frequent co-authorship among members. This indicates well-established research networks and potentially high productivity in terms of publications. Overall, the VOSviewer visualization provides valuable insights into the collaborative landscape of PPRV and PPR research, showcasing key researchers, geographic diversity, and the interconnected nature of global research efforts. This analysis helps identify potential collaborators and understand the collaborative dynamics within the PPRV and PPR research community, supporting the goal of eradicating PPR globally.

Advanced research countries partner with heavily affected regions to test and distribute vaccines, exemplified by initiatives under the PPRV Global Eradication Program, which aims to eradicate the disease by 2030. Our findings, illustrated in Figure 2, underscore the substantial contributions from the UK, France, and China, alongside Indian and other international research institutions, in advancing the understanding and management of PPR. This international collaboration must include capacity-building and training programs to enhance the skills of researchers and veterinary professionals in developing countries [18]. Multiple international agencies should cooperate to increase funding for PPRV and PPR research, ensuring efficient resource allocation and avoiding duplication of efforts. The scientific publications resulting from these collaborations disseminate research findings, fostering innovation and highlighting the collaborative nature of PPRV and PPR research.

The VOSviewer visualization (Fig. 3) highlights the importance of PPRV and PPR in the context of small ruminant diseases, virology, and genetics. The dense interconnections between keywords related to animals and disease suggest a robust network of research focusing on the epidemiology, diagnostics, and impact of PPR. However, to achieve the eradication of PPRV, several critical areas require attention beyond those highlighted. Robust surveillance and monitoring systems are essential for early detection and tracking of outbreaks [19]. The reverse transcription polymerase chain reaction (RT-PCR) technique is the preferred method for virus detection for reasons of sensitivity and specificity, and detecting PPRV from all four lineages of the virus does not appear in the hotspot keywords [5]. Besides, consideration of the use of vaccines; strategic vaccination was the main aspect of PPR eradication and also did not appear in the hotspot keywords. Research efforts should focus on developing and disseminating more effective vaccines, understanding transmission dynamics, and identifying potential wildlife reservoirs. Additionally, improve and implement RT-PCR as a diagnostic tool for epidemiological detection of PPRV lineage [20].

Authors tend to publish their research in journals with a scope on infectious diseases posing major economic threats, focusing on diagnosis, prevention, management, and outbreak communication. This includes Transboundary and Emerging Diseases and Tropical Animal Health and Production. Besides, basic science journals like the Journal of Virological Methods and Viruses have scope for novel and comprehensively tested methods in virology, including viral components, diagnostics, replication, transmission, vaccines, and antivirals. The prominence of the research output in these journals reflects the interdisciplinary nature of PPR and PPRV.

While this study provides valuable insights into the global research trends on PPRV and PPR, it has several limitations that should be addressed in future analyses. The study is limited by its reliance on a single database, Scopus, which may overlook relevant research published elsewhere or in non-English languages. Additionally, the exclusion of conference papers, editorials, and non-peer-reviewed publications could result in missing early or innovative findings. The focus on publication volume rather than research quality and impact may also skew the interpretation of research productivity. Furthermore, while the study highlights key research gaps such as vaccine development and diagnostic methods, it does not delve deeply into the practical challenges of implementing these solutions, especially in resource-poor regions. Finally, although the study maps author collaborations, it lacks a detailed exploration of how these networks contribute to real-world outcomes, such as the success of eradication programs. Addressing these limitations through a more inclusive, qualitative, and multi-database approach would provide a more comprehensive understanding of PPRV research and its implications for disease eradication efforts.

## Conclusion

In conclusion, the findings underscore the increase in global scientific production resulting from international collaboration on PPRV and PPR research during the past two decades. The main topic was the emergence of PPRV and PPR in small ruminants. The identified research gaps call for further cooperation between developed countries and North African and Middle Eastern countries where the disease is endemic. To improve the use of RT-PCR in the longitudinal epidemiology of PPR, research hotspots should focus on making vaccines, running vaccination campaigns, and building up the skills of veterinarians. The study’s findings can help policymakers, funders, and researchers decide which resources and strategies are most important for managing and getting rid of PPRV, which will protect the health of livestock and keep the economy stable. Addressing these gaps will contribute to the development of more effective PPR eradication worldwide.

## List of abbreviations

CIRAD, French Agricultural Research Centre for International Development; FAO, Food and Agriculture Organization; ICAR, Indian Council of Agricultural Research; INRAE, Institut National de Recherche pour l’Agriculture, l’Alimentation et l’Environnement; PPR, peste des petits ruminants; PPRV, peste des petits ruminants Virus; RT-PCR, reverse transcription polymerase chain reaction; WOAH, World Organisation for Animal Health; VOSviewer, visualization of similarities viewer.
